# Schools That Promote the Improvement of Academic Performance and the Success of All Students

**DOI:** 10.3389/fpsyg.2019.02920

**Published:** 2020-02-07

**Authors:** Pilar Arnaiz-Sánchez, Remedios de Haro, Salvador Alcaraz, Ana Belén Mirete Ruiz

**Affiliations:** Department of Didactics and School Management, University of Murcia, Murcia, Spain

**Keywords:** academic performance, effective schools, educational improvement, intervention program, quasi-experimental design, inclusive education

## Abstract

The movement for effective schools and school improvement has enjoyed a long history, at both the theoretical and practical level. The contextual variables focused on the educational process of the classrooms have been identified in numerous investigations, concluding that the improvement of academic performance is a key element of the movement. The main objective of this research is focused on verifying the effectiveness of the treatment based on collaborative/cooperative learning methodologies and projects to improve the linguistic and mathematical competence as an enhancing element of academic performance. The sample consists of 228 students belonging to two public schools located in the city of Murcia (Spain), selected through judgmental or discretionary sampling. A quasi-experimental design with pretest and post-test and control group was employed, verifying the effectiveness of the treatment, and how it influences the improvement of the academic performance of the students in the experimental group. It concludes by pointing out the importance of learning strategies and applied teaching methodologies, understanding both within the conglomerate of process factors in the improvement of academic performance.

## Introduction

An objective present in every government agenda is ensuring the development of an inclusive, equitable, and quality education that promotes lifelong learning opportunities. UNESCO (2015), in its enactment of the 2030 Agenda for Sustainable Development, proposes as one of its fundamental objectives the promotion of this type of education in order to promote success for all, as well as good levels of academic performance. To do this, schools must develop the necessary conditions and processes that realize this goal so that schools become “inclusive and effective learning environments within the framework of a school for all” (Arnaiz, 2012, p. 31).

Inclusive education is connected to the movement for effective schools and school improvement, making it clear that what happens in classrooms, in terms of organization, interventions, activities proposed by teachers, among other factors, has a critical value for the improvement of academic performance and success for all students, in short, for the attainment of expected educational achievement (Rutter et al., 1979; Brophy and Good, 1986; Mortimore et al., 1989; Davis and Thomas, 1992; Reynolds and Cuttance, 1992; Scheerens, 1992; Ramasut and Reynolds, 1993; Teddlie and Stringfield, 1993; Creemers, 1994; Reynolds et al., 1994; Castejón, 1996; Wyatt, 1996; Murillo, 2003a, b; Arnaiz et al., 2018).

An effective school is one that “achieves the comprehensive development of each and every one of its students, greater than would have been expected when considering their previous performance and the social, economic and cultural situation of their families” (Murillo, 2005, p. 25). The fundamental role of the school in improving academic performance, as considered by the movement for Effective Schools and Inclusive Educational, calls into question the decisive characteristic given to some variables in academic performance, such as the socioeconomic origin of families. An emblematic example of this was the Coleman (1968), which rejected any possibility of improvement and overcoming inequalities. Fifty years after its publication, research has shown that a school, through its interventions, procedures and actions, can promote the overcoming of inequalities and, therefore, facilitate educational success. In this line of argument, Flecha and Buslon (2016); Madigan et al. (2016), Marqués (2016); Muro et al. (2018), among others, indicate that successful educational interventions produce significant improvements in academic results, overcoming the determinative and deficient views present in said report.

Traditionally, the improvement of student academic performance has been focused on three main components: personal characteristics or individual factors (such as intelligence), contextual factors or improvement of the educational environment (such as school improvement) and factors related to one’s self-beliefs, understanding of self, and the environment (such as responsibilities, mentality, and personal experiences) (Van Mieghem et al., 2018). In other works, procedural factors such as school environment or leadership have been taken into account. Along the same lines, the works of Murillo (2007) and Jornet et al. (2012) classify these factors as entry (gender, socio-economic level, mother tongue, school resources, etc.), process (study habits, academic expectations, family support, school environment, teaching methodology, etc.) and product (academic performance). In turn, these factors can be divided into two areas or levels: students and educational centers.

Other authors have identified multiple individual components, such as cognitive ability (Lu et al., 2011), self-perspective (Miñano et al., 2012; Dedrick et al., 2015), gender (Miralles et al., 2012), expectations (Zimmerman et al., 1992; Miller et al., 1993), socioeconomic status (Miralles et al., 2012), opportunities for physical activity (Takehara et al., 2019), and motivation (Castejón et al., 2016; Muro et al., 2018), as predictive factors in academic performance. Likewise, Escarbajal et al. (2019) and Pulido and Herrera (2019) identified family status, cultural origin, and age as influential variables on the issue at hand. According to these authors, students of immigrant origin obtain lower academic results in relation to students of native origin.

Ruiz-Esteban et al. (2018) relate academic performance to academic goals and motivational patterns. Similarly, Rodríguez and Guzmán (2019) highlight the influence of academic goals and the variables of environment, support, and socio-labor status of families on academic performance.

Contextual variables have also been highlighted as important predictive factors in academic performance (Jeynes, 2010; Zuffianò et al., 2013). Notable among them is the orientation to learning goals (Hsieh et al., 2007), learning strategies (Preckel and Brunner, 2015; Veas et al., 2017), popularity (Schwartz et al., 2006), adaptation to the school context (McCoach and Siegle, 2003), early attention (Franco et al., 2017), the use of methodologies such as peer mentoring (Durán and Vidal, 2004; Dunn et al., 2017), the participation of families in the school (Wilder, 2014), or the relationships established between students and teachers (Ramberg et al., 2019).

Hincapie et al. (2018) relate academic performance to teaching methodology. In this case, good results are obtained when problem-based learning is used to improve academic performance and critical thinking. Likewise, Karrera et al. (2019) highlight the importance of using teaching strategies such as project work to improve academic performance in Primary Education. Similarly, the relationship between access to, and the use of, information and communication technologies and academic performance has been studied in depth. Various studies indicate an increase in academic performance in those schools where the use of these technologies is utilized (García-Martín and Cantón-Mayo, 2019; Hinojo et al., 2019). Several authors (Molina-López et al., 2018) have even pointed out that competition between schools shows a positive effect on the academic performance of students.

Other studies show empirical evidence of the influence of some variables on academic performance such as the mean socio-economic and cultural level of the school (Perry and McConney, 2010a, b), school size or teacher–student ratio (Nath, 2012), as well as school process factors such as grouping students according to their academic ability (Meunier, 2011; Kunz, 2014), teaching methodology (Nath, 2012; Payandeh-Najafabadi et al., 2013), or the learning environment (Payandeh-Najafabadi et al., 2013; Santos et al., 2013), among others.

Given the great diversity of factors influencing the academic performance shown in the scientific literature, in this article, we focus attention on some variables – teaching methodologies – that influence the academic performance of the students participating in a particular school. To that end, the recommendation made by Miralles et al. (2012) has been followed, which urges experience studies to be carried out that allow us to understand which factors contribute to the effective functioning of schools from the point of view of student academic performance.

This work is immersed in one of those schools that work daily to be effective in responding to the goals set in order to offer inclusive and quality education and thus improve the academic performance of all students by overcoming potential inequalities. This is due to the commitment of teachers to improve the quality of teaching-learning processes in the center and guarantee the principle of equity for all students. Being a center located in a marginal neighborhood, socially and economically deprived, with families experiencing unemployment and other vulnerable circumstances, the academic level of the students was very low. This situation leads to those families who have the possibility of not sending their children to this center, but to other schools in the area, will do so. This school thereby becomes marginal. The teachers set out to change this situation through innovation and improvement actions (included in the Center Educational Project) and are essentially focused on the areas of Language and Mathematics.

Explicitly, the purpose of this contribution is to analyze the improvement in student academic performance after introducing a series of changes in relation to the curriculum – linguistic and mathematical competence – and teaching–learning strategies used by teachers and students. In short, we are assessing the ability of variables focused on the educational process in the classrooms – didactic strategies – for the improvement of students’ academic performance.

As has been shown, there has been in-depth analysis of academic performance in research with personal and social variables, but there are fewer studies focused upon school variables in vulnerable contexts. In this way, the questions that guide this research are as follows: Can didactic strategies improve the academic performance of students in these contexts? Can an educational intervention treatment based on collaborative/cooperative learning and project work improve the performance of students in vulnerable contexts in the areas of language and mathematics?

## Materials and Methods

### Participants

The population under study is composed of Primary Education students (6–12 years old) belonging to two public schools located in an urban area 7 km away from the city of Murcia (Spain). As the management team informed, their families’ socio-economic and cultural level is medium–low and they mostly work in the service sector. The expectation of families regarding the education of their children is centered on their children receiving a standard education, and only a small proportion of the parents aspire for their children to engage in higher studies.

Both schools have similar characteristics, are state run, and are accountable to the Ministry of Education. The school where the experimental group is based has 20 units, 6 of Infant Education and 14 of Primary Education, which represents a total of 476 students, distributed in the stages of Infants (148 students) and Primary (328 students). The control group school has 15 units, 5 of Infant Education and 10 of Primary Education, which represents a total of 367 students, 135 in Infants and 232 in Primary Education.

Discretionary non-probabilistic or judgment sampling was used (McMillan and Schumacher, 2005; Hernández-Pina and Maquilón, 2010), dependent on knowledge of the characteristics of both schools as well as their population. Finally, the actual sample was composed of 228 students, which, based on a total population of 560 students, is considered a 95% confidence level with 5% sample error (calculation made via surveymonkey.com).

Of this sample, 130 (57.01%) were part of the control group and 98 (42.99%) were part of the experimental group. Regarding its distribution by gender, 137 (60.1%) participants were boys, and the remaining 91 (39.9%) were girls, without knowledge of the sexual identity of any of the participants. The description of the participants is included in Table 1.

**TABLE 1:** Distribution of the participants.

Center	Experimental	Control	Total
**Gender**			
Male	60(61.2%)	77(59.23%)	137
Female	38(38.2%)	53(40.77%)	91
Total	98(100%)	130(100%)	228

### Measure

The measure used was the average score obtained in the assessment of language and mathematics in the first trimester, as well as those in the final evaluation in both centers (Table 2). These evaluations were not specifically designed for this project in respect of the performance and criteria of each of the centers, since both use the learning standards established in educational regulations at the national and regional level.

**TABLE 2:** Description of the measurement process.

Experimental group	1st-trimester average score (linguistics + mathematics)	Treatment	Final average score (linguistics + mathematics)
Control group	1st-trimester average score (linguistics + mathematics)		Final average score (linguistics + mathematics)

### Procedure

The informed consent of the parents for the participation of the students in the proposed program was requested by the management team of the center by means of a signed letter. The program was carried out in the experimental group as a study/work activity in the center for the development of the curriculum in Language and Mathematics in which all students participated.

Both groups (experimental and control) used the same series of textbook for the development of linguistic and mathematical competencies (Ibarrola, 2017) covering first to sixth grade of Primary education. In addition, in the experimental group, the use of the textbook was combined with the program specifically designed to improve academic performance in language and mathematics.

Once the existing levels in Language and Mathematics of both groups, control and experimental, were established, the intervention procedure for experimental group was designed. This was focused on the process factors because they are the ones that have a significant impact on the academic performance of the students. In this sense, the objectives to be achieved were established and activities were designed according to the needs and characteristics of the students in order to improve their skills. In the same way, the teaching methodology and the didactic strategies, which most encouraged the students’ motivation and confidence toward learning, were established. To do this, we turned to cooperative learning, peer tutoring, and discovery learning methods that connect the curricular content with real-life experiences and the interests of the students, carrying out small research projects and problem-solving tasks (problem-based learning). In addition, the intervention prioritized that the teacher–student interaction be characterized by a safe and close environment.

Therefore, the treatment carried out for the improvement of academic performance consisted, in general, in the realization of a series of activities differentiated by courses. In the linguistic area, students presented suggestions for poems, short texts, newspaper articles, stories, comics, puns, oral expression exercises, etc., turning the classroom into a creative literary workshop with the aim of integrating the development of skills and multiple intelligences. In the mathematical area, the work undertook highlighted the development of logical reasoning, using puzzles and specific mathematical challenges for each cycle.

The time allocated to the intervention was three weekly sessions each of 45 min, from January to June. The coordination and development of each session as well as the final evaluation of the students fell to each of the classroom tutors.

In the case of the control group, the dynamics of the learning process undertaken up to that point in the areas of language and mathematics were not changed, consisting of reading through the lessons, teacher explanation, and carrying out the exercises (Ibarrola, 2017).

### Design and Data Analysis

A quasi-experimental design with pretest and post-test and control group was adopted (Hernández-Pina and Maquilón, 2010; Campbell and Stanley, 2015). This type of design allows comparison between a group that has received intervention, called an experimental group, and another one called the control group to which no modification in the educational process has been applied. It is one of the most commonly used designs in socio-educational research because it does not require a random assignment of participants, but allows for the attainment of balanced groups.

Given the characteristics of the design used, the independent factor or variable belonged to one or the other group (experimental or control), while the criterion or dependent variable was the mean of the academic performance of the subjects (Williams, 1952; Box, 1971; Berenblut and Webb, 1974), obtained in the evaluations in the areas of mathematics and language.

For data analysis, the general linear model (GLM) of repeated measures has been used in order to assess the effectiveness of the treatment, through which, groups of related dependent variables that represent different measures of the same attribute are analyzed (Freeman, 1973; Bryant and Paulson, 1976; Wood et al., 1978; Vuchkov and Solakov, 1980; Defeo and Myers, 1992). To carry out the data analysis, the statistical package SPSS, version 21.0 was used.

The researcher must be conscious of the ethical responsibility involved in the conduct of an investigation, especially when it deals with human beings (McMillan and Schumacher, 2005). In this way, following the principles and norms published by the American Psychological Association [APA] (2010), the rights and dignity of the participants were guaranteed at all times in this investigation; this was endorsed by the favorable report issued for the realization of this research by the Research Ethics Commission of the University of Murcia.

## Results

The objective of this work is focused on verifying the effectiveness of the treatment of improving linguistic and mathematical competences as a favorable element of academic performance at a general level. In order to respond to this, our first step was to ensure the similarity of the experimental and control groups before the intervention; therefore, a comparative analysis of means was performed in the pretest phase, verifying that there were no statistically significant differences between these groups (Table 3).

**TABLE 3:** Test *t* of mean difference. Pretest.

	M Experimental	dt	M Control	dt	*p*	*t*	gl
Academic performance	6.00	1.86	5.66	1.94	0.18	1.35	229

The analysis of the sample demonstrated that the populations are distributed normally. The Box M test was applied, obtaining non-homogeneous variance–covariance matrices (*F* = 13,561; gl = 5749165.693 and *p* < 0.001), but, since the groups are approximately the same size [according to Hair et al. (1999), the size of the largest group divided by the size of the smallest group should be less than 1.5] and the highest variance ratio between the groups does not exceed the 10:1 ratio considered as the maximum limit in the analysis of profiles for Tabachnick and Fidel (2007), the violation of this assumption has a minimal impact.

To evaluate the effectiveness of the treatment in the sample, a GLM of repeated measures was also used. The dependent variable was the mean academic performance. Gender was included as covariate, obtaining a non-significant interaction. This implies that there is a significant effect of the intervention program on the academic performance of the experimental group, while the covariate was not significant.

Intra-subject factors are represented in the evaluation times (pre- and post-test) for the dependent variable. Inter-subject factors depended on the presence or absence of the treatment (i.e., the experimental group or the control group).

As shown in Table 4, the effects of the intra-subject and inter-subject test show that the effect of the interaction between the time of the evaluation (pretest and post-test) and the implementation of the program of activities is significant (*p* < 0.001). In addition, the observed power (the correct rejection of the null hypothesis of equality of means) is optimal.

**TABLE 4:** Summary of intra-/inter-subject analysis.

Source	Type III	gl	*F*	*p*	η^2^ partial	Obs. power
**Academic achievement**						
Intra	0.863	1	0.902	0.343	0.004	0.157
Intra × gender	0.367	1	0.384	0.536	0.002	0.095
Intra × inter	70.195	1	73.384	0.000	0.246	1.000
Error intra	215.224	225				
Inter	143.635	1	23.824	0.000	0.096	0.998
Error inter	1356.512	225				

The effect size (η^2^), that is to say, the proportion of the total variability attributable to a factor (or the magnitude of the difference between one time and another, as a result of the interaction between the moment of evaluation and the application of the program), obtains the best results when the interaction is analyzed according to the experimental and control group, reaching values of 0.246.

**FIGURE 1:**
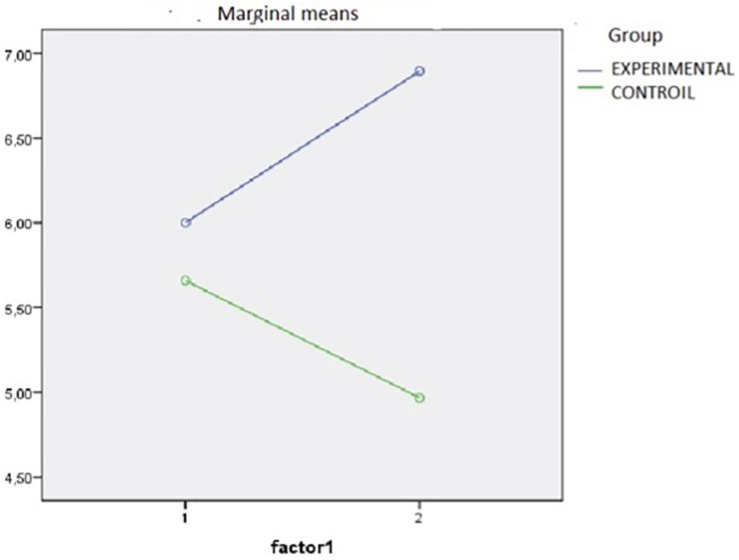
Graphical representation of the means of academic performance of the two groups in pre- and post-test time.

Figure 1 presents the interaction graph, which illustrates the directions of the differences. The total score of the academic performance of the experimental group was significantly higher once the intervention program was completed, the mean score being 6.90 (SD = 2.03), while it was 4.97 (SD = 1.65) for the control group.

Finally, it should be noted, in the analysis of the means obtained by both groups, that in each case statistically significant differences were found after the Student’s *t* test for related samples, although in the case of the experimental group, as observed in Figure 1, they were increased (*p* < 0.001), while for the control group, the mean values decreased (*p* < 0.001).

## Discussion and Conclusion

The results presented above demonstrate the value and effectiveness of the program used given the good results obtained and the improvement in the academic performance of the participating students compared to those who did not benefit from the advantages of the said program. Since the main objective of this work was to verify the effectiveness of the implementation of an intervention program based on cooperative learning, peer tutoring, and problem-based learning, it has been possible to verify that the final scores have risen in the experimental group by almost 1 point, based on a global average score from 6.00 points before treatment, to a final overall average score of 6.90 points after the application of the program.

In this sense, we agree with other studies that highlight the importance of learning strategies (Preckel and Brunner, 2015; Dunn et al., 2017; Hincapie et al., 2018; Karrera et al., 2019), or the teaching methodology used (Nath, 2012; Payandeh-Najafabadi et al., 2013) in the improvement of academic performance. Consequently, we are able to demonstrate how important it is for the school to improve performance and overcome inequalities as indicated by Ainscow (2005), Arnaiz et al. (2018), Flecha and Buslon (2016), Madigan et al. (2016), and Murillo (2005).

The school where the intervention program was developed had the goal of developing the necessary processes to promote quality education so that the students’ language and math skills improved; desired to be more and more effective in achieving this end, as indicated by different experts, this is a basic requirement in achieving success for everyone and, consequently, good levels of academic performance (Murillo, 2008; Arnaiz, 2012; Van Mieghem et al., 2018).

This work has empirically proven the effectiveness of the implementation of a program for the improvement of skills in terms of academic performance. This program could be the start of a cycle of constant improvement, where the performance factor is eclipsed by other types of elements, such as the collective atmosphere of pursuing the enrichment of the teaching and learning processes, or the wider social recognition of the school’s efforts (Murillo, 2007; Jornet et al., 2012).

Ramberg et al. (2019) established the relationship between student and teacher as a performance-enhancing element. We believe that teacher satisfaction with innovations aimed at the improvement of educational quality, as well as the acceptance of common goals with the school, could be a differentiating element to take into account in future work. In this sense, we do not want to lose the opportunity to point out some of the contributions that have been given to us by professionals involved in the implementation of the program, and collected in our field notebooks during some of the conversations held with the teaching staff, which appear to verify, subjectively, those data that we presented previously.

“My personal assessment is that students enjoy playing with language, having fun and making us participants in their compositions, which in many cases are extraordinarily original, allowing the transmission of this poetic flow to their future school studies” (5th grade teacher of Education Primary).

“The overall assessment of the work done with the students of the 5th and 6th grade of primary school has been highly satisfactory” (6th grade Primary Education teacher).

“Throughout the course we have been able to constantly enjoy inventing, creating and recreating to find appropriate learning processes that will take us away from routine and preconceived ideas” (5th grade Primary Education teacher).

In spite of the good results obtained, it is possible to deepen the research carried out by incorporating new study variables, which would allow advancement in the identification of the factors that promote the improvement of academic performance and, consequently, the development of effective schools. In the same way, it would be interesting to use other measurement tests in order to access the improvement of student performance – different to teacher grading – such as standardized tests in order to eliminate elements of subjectivity or equivalence between teachers, classrooms, and educational centers (McCoach and Siegle, 2003). Together with this, new variables could be incorporated to measure the effectiveness of the programs used, an element such as students in disadvantaged contexts, to show if successful actions improve academic results and consequently contribute to overcoming inequalities, such as indicated by Flecha and Buslon (2016).

Ultimately, the study carried out indicates the importance of learning strategies and applied teaching methodologies, understanding both within the conglomerate of process factors in the improvement of academic performance (Brophy and Good, 1986; Davis and Thomas, 1992; Reynolds and Cuttance, 1992; Scheerens, 1992; Ramasut and Reynolds, 1993; Creemers, 1994; Reynolds et al., 1994; Castejón, 1996; Wyatt, 1996; Murillo, 2003a,b). Hence, obviously, we want to point out the importance of what we found and the implementation of it in other educational centers in order to promote effective schools capable of offering a quality, equitable, and inclusive education for all, as UNESCO (2017) reminds us.

Finally, it is possible to express the limitations present in this study, which reside in the fact that only two curricular subjects or areas (language and mathematics) have been taken into account, ignoring others that are equally relevant in the measurement of academic performance. Another aspect to consider could be the application of a retest to verify the effectiveness of the implemented program, although this would take on greater importance if the experience were isolated as an anecdotal implementation of the program. Similarly, the participation of only two centers limits the extrapolation of results obtained to other contexts with similar characteristics. All this being said, it invites us to carry out new studies that overcome these limitations and promote educational improvement in this and other educational centers.

## Data Availability Statement

The data may be consulted by making an express request to the authors through the following email: mailto:parnaiz@um.es.

## Ethics Statement

The studies involving human participants were reviewed and approved by the Research Ethics Commission of the University of Murcia. The participants provided their written informed consent to participate in this study.

## Author Contributions

The study was carried out collaboratively, both in the development of the project and distribution tasks, although we found specific tasks had to be defined at different moments of the process. PA-S coordinated and led the group, in addition to revisions and corrections of text, analysis of data, discussion, and presentation of conclusions. SA and RH were directly responsible for the theoretical section, as well as for subsequent revisions and corrections. AM was responsible for data analysis and presentation of results.

## Conflict of Interest

The authors declare that the research was conducted in the absence of any commercial or financial relationships that could be construed as a potential conflict of interest.
